# Cross‐scale effects of spruce budworm outbreaks on boreal warblers in eastern Canada

**DOI:** 10.1002/ece3.4244

**Published:** 2018-06-27

**Authors:** Mark C. Drever, Adam C. Smith, Lisa A. Venier, Darren J.H. Sleep, David A. MacLean

**Affiliations:** ^1^ Canadian Wildlife Service Environment and Climate Change Canada Delta British Columbia Canada; ^2^ Canadian Wildlife Service Environment and Climate Change Canada National Wildlife Research Centre Ottawa Ontario Canada; ^3^ Canadian Forest Service Natural Resources Canada Great Lakes Forestry Centre Marie Ontario Canada; ^4^ National Council for Air and Stream Improvement Inc. Montreal QC Canada; ^5^ Faculty of Forestry and Environmental Management University of New Brunswick Fredericton New Brunswick Canada

**Keywords:** Atlantic Canada, breeding bird survey, defoliation, forest birds, hierarchical Bayesian model, numerical response, Ontario, Quebec, spruce budworm outbreaks

## Abstract

Insect outbreaks are major natural disturbance events that affect communities of forest birds, either directly by affecting the food supply or indirectly by changing the vegetation composition of forest canopies. An examination of correlations between measures of bird and insect abundance across different spatial scales and over varying time lag effects may provide insight into underlying mechanisms. We developed a hierarchical Bayesian model to assess correlations between counts of eight warbler species from the Breeding Bird Survey in eastern Canada, 1966 to 2009, with the presence of spruce budworm (*Choristoneura fumiferana* Clem.) at immediate local scales and time‐lagged regional scales, as measured by extent of defoliation on host tree species. Budworm‐associated species Cape May warbler (*Setophaga tigrina*), bay‐breasted warbler (*Setophaga castanea*), and Tennessee warbler (*Oreothlypis peregrina*) responded strongly and positively to both local and regional effects. In contrast, non‐budworm‐associated species, Blackburnian warbler (*Setophaga fusca*), magnolia warbler (*Setophaga magnolia*), Canada warbler (*Cardellina canadensis*), black‐throated blue warbler (*Setophaga caerulescens*), and black‐throated green warbler (*Setophaga virens*), only responded to regional effects in a manner that varied across eastern Canada. The complex responses by forest birds to insect outbreaks involve both increased numerical responses to food supply and to longer term responses to changes in forest structure and composition. These effects can vary across spatial scales and be captured in hierarchical population models, which can serve to disentangle common trends from data when examining drivers of population dynamics like forest management or climate change.

## 1 INTRODUCTION

Outbreaks of eastern spruce budworm (*Choristoneura fumiferana* Clem.) are major natural disturbance events affecting long‐term cycles of growth and succession in forests of eastern North America (Baskerville, 1975). Adult budworm moths lay their eggs on the foliage of host trees in midsummer, and the larvae overwinter in silken hibernacula, from which they emerge the following spring to feed on the new foliage. During epidemic conditions, consecutive defoliations by budworm can result in widespread tree mortality occurring 4–5 years after first defoliation (MacLean, 1980). Such outbreaks can affect hundreds of thousands of hectares of its host tree species (Simpson & Coy, 1999), primarily balsam fir (*Abies balsamea*), white spruce (*Picea glauca*), red spruce (*Picea rubens*), and black spruce (*Picea mariana*). Thus, spruce budworm is the most important of North America's native forest defoliators (Hardy, Mainville, & Schmitt, 1986; MacLean, 2016).

The ecological interplay between spruce budworm and insectivorous forest birds has long interested ecologists (Kendeigh, 1947; MacArthur, 1958). Spruce budworm outbreaks have strong effects on forest bird communities (Venier & Holmes, 2010) and can result in large increases of overall bird densities, up to two to five times the abundances observed in preoutbreak conditions (Holmes, Sanders, Fillman, & Welsh, 2009; Kendeigh, 1947; Morris, Cheshire, Miller, & Mott, 1958; Sanders, 1970; Venier, Pearce, Fillman, McNicol, & Welsh, 2009). Some bird species have very clear positive responses to the local density of spruce budworm that are consistent across many studies, including Cape May warbler (*Setophaga tigrina*), bay‐breasted warbler (*Setophaga castanea*), and Tennessee warbler (*Oreothlypis peregrina*) (Baltz & Latta, 1998; Rimmer & McFarland, 2012; Venier, Holmes, & Williams, 2011). The responses of other species are less clear and depend on the location and the spatial and temporal scales of the study, for example, Blackburnian warbler (*Setophaga fusca*) and Canada warbler (*Cardellina canadensis*). Local effects primarily occur as birds respond to the superabundant food supply provided by the budworm larvae (Venier & Holmes, 2010). Indirect effects are also possible, as budworm defoliation opens the forest canopy and results in changes in competitive interactions as the bird community responds to the outbreak.

Previously, studies examining dynamics of bird abundance as the local density of budworms vary over time have been conducted at local scales (e.g., 10 km^2^ study site); or at broad regional scales (e.g., provincial, national, or even continental), examining correlations between indices of budworm and bird abundances from large monitoring programs (Bolgiano, 2004; Patten & Burger, 1998; Sleep, Drever, & Szuba, 2009). However, estimating population‐level responses of forest birds to budworm outbreaks, which is necessary for effective bird species management, is complicated. Broadscale studies may be confounded by the potential for spurious correlations due to coinciding temporal trends in bird populations and budworm abundance (Venier, Holmes, Pearce, & Fournier, 2012). At local scales, scaling local estimates into population‐level inferences may be difficult due to variance in responses of forest birds to outbreaks through regional variation in forest composition, climate variability, and management practices (Grinde et al., 2017; Rauchfuss & Svatek Ziegler, 2011). Therefore, studying relationships between budworm and forest birds simultaneously at multiple scales will allow us to estimate both local responses and regional responses after controlling for local response, and thereby derive conclusions that may be directly relevant for managing regional, national, and continental populations of warblers on their breeding grounds. In addition, a more general understanding of how disturbance events affect forest biodiversity is an essential step in forest management that seeks to maintain nontimber values and confer ecological resilience to managed forests through the emulation of natural disturbance regimes (Bergeron, Chen, Kenkel, Leduc, & Macdonald, 2014; Drever, Peterson, Messier, Bergeron, & Flannigan, 2006).

Budworm‐associated warbler species have strong local responses to outbreaks through potential mechanisms that reflect both movements of birds into areas affected by budworm as well as local increases in populations as the superabundant food resource leads to increased reproductive rates (Holmes et al., 2009). After an outbreak begins to subside and tree mortality eventually reduces available food for the insect, abundance of budworm‐associated species should also increase in neighboring areas not directly affected, as the increased population redistributes to suitable habitat across the broader landscape. These ecological processes suggest that population‐level effects of budworm on forest birds should occur at two distinct spatial and temporal scales: Species should respond at local scales in a given year (higher territory density from preferential habitat selection) and at a broader, regional scale after a few years of sustained outbreak (increased reproductive rate followed by dispersal as local outbreaks decline). If increased recruitment at the site of the outbreak leads to increased dispersal to areas beyond the outbreak, then regional‐scale responses could be observed on lags as short as 1 year. However, in the early years of an outbreak, local density of budworm stays relatively high and so the increasing warbler populations are likely under little pressure to redistribute across the landscape. Therefore, the regional‐scale effects are likely much stronger after four or 5 years of sustained outbreak, when the local abundance of budworm has started to decline (Holmes et al., 2009).

Spruce budworm outbreaks are currently occurring in eastern Canada, with over 7.2 million hectares of defoliation in Quebec in 2017 (QMFFP, 2017), and populations building in the Atlantic Provinces (Healthy Forest Partnership, 2017). To better understand bird species’ relationships with budworm at both local and regional scales simultaneously, and to provide important estimates of expected broadscale population responses, we assessed correlations between warbler abundance and measures of incidence of spruce budworm at multiple spatial and temporal scales, using datasets that span most of the budworm‐affected forests of eastern Canada over a >40‐year period, 1966–2009. We used a hierarchical Bayesian model to assess correlations between counts of warblers and the geographic extent of spruce budworm locally in each year and regionally at a lagged time‐scale. We used bird count data from the Breeding Bird Survey (BBS) and budworm data measured from aerial surveys conducted by Canadian Provinces as the area of defoliation on host tree species. We compared responses of eight Parulid warbler species, including three species with known associations with spruce budworm and five others that have shown mixed responses among previous studies (positive, negative, or no response). We predicted that if a warbler species can track budworm availability in the region and the hyperabundant food resource leads to increases in their population, then we should expect both a positive local response to budworm in a given year on a given BBS route and a regional response after a short time lag reflecting new recruits to the population that are dispersing out of the budworm‐affected areas.

## 2 METHODS

### 2.1 Analytical strata

**Figure 1:**
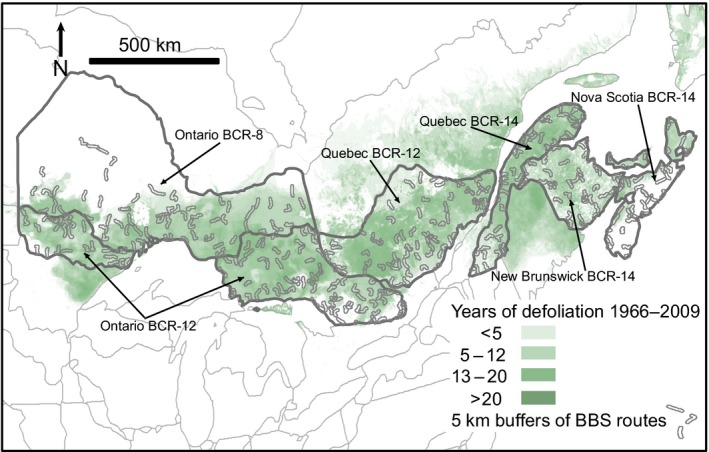
Map of eastern Canada identifying the geographic strata used, locations and number of Breeding Bird Survey (BBS) routes, and areas affected by moderate to severe spruce budworm defoliation attributed to spruce budworm, 1966 to 2009

Our analysis was structured on the same strata (geographic regions) used to estimate population status and trends from the BBS data in Canada (Smith, Hudson, Downes, & Francis, 2014). These strata are defined by intersections of Bird Conservation Regions with provincial and territorial borders (e.g., the portion of BCR‐12 that overlaps Ontario). The six strata overlapped most of the extent of the spruce budworm outbreak that occurred in Eastern Canada in the 1970s and 1980s (Gray & MacKinnon, 2006; Figure 1). The Quebec‐BCR‐8 stratum was excluded because the BBS routes in this region have not been surveyed a sufficient number of times to meet our minimum BBS data criteria (below). The final data set included defoliation and BBS data from six analytical strata in Eastern Canada (Figure 1).

### 2.2 Breeding bird survey data

We used observations collected on Canadian BBS routes from 1966 through 2009, which included counts of the number of birds observed on routes that were surveyed under suitable weather conditions (Sauer et al., 2012). For each species, only routes on which the species had been observed at least once were included. In addition, for each species, strata were included if they overlapped the area affected by spruce budworm outbreaks in eastern Canada (Gray & MacKinnon, 2006; Figure 1), and the species had been observed on at least three routes and in at least 4 years/route, averaged across the included routes.

Responses to spruce budworm defoliation were compared for eight warblers. These warblers included three species that have known positive relationships with spruce budworm (Baltz & Latta, 1998; Rimmer & McFarland, 2012; Venier et al., 2011), Cape May warbler, bay‐breasted warbler, and Tennessee warbler; three species that may potentially respond to budworm outbreaks (Venier & Holmes, 2010), Blackburnian warbler, magnolia warbler (*Setophaga magnolia*), and Canada warbler (Sleep et al., 2009; but see Venier et al., 2012); and two species with no known association with spruce budworm (Holmes, Rodenhouse, & Sillett, 2005; Morse & Poole, 2005), black‐throated blue warbler (*Setophaga caerulescens*), and black‐throated green warbler (*Setophaga virens*).

### 2.3 Budworm defoliation

Maps of forest areas defoliated by spruce budworm were obtained from the annual surveys of forest insect damage from fixed‐wing aircraft conducted by each of the Canadian Provinces (e.g., QMFFP, 2017). Aerial defoliation surveys were typically conducted during about a 2‐week period in late July or early August, immediately following the completion of spruce budworm feeding. At this time, a distinct reddish‐brown coloration of dry foliage appears, as a result of budworm severing and webbing together needles in the process of feeding (MacLean & MacKinnon, 1996). In‐flight surveyors delineate polygons of insect damage using topographic maps and assign a severity class to an estimate of current‐year defoliation to each polygon (MacLean & MacKinnon, 1996; Candau, Fleming, & Hopkin, 1998; Gray, Régnière, & Boulet, 2000). Areas with noticeable reddish‐brown coloration of foliage were typically recorded on maps in light (11%–30%), moderate (31%–70%), and severe (71%–100%) defoliation classes (e.g., Carter & Lavigne, 1993). Areas with no noticeable defoliation were assigned to a nil (0%–10%) class. Aerial sketch mapping of spruce budworm defoliation is a viable technique that can be used for both surveys and forest management applications (MacLean & MacKinnon, 1996; Taylor & MacLean, 2008).

For these analyses, we used spatial polygons that represented the areas affected by moderate or severe defoliation (31%–100%) by spruce budworm in each year from 1966 through 2009, as these are indicative of high budworm populations. Annual defoliation less than 30% has little effect on tree growth or mortality (Erdle & MacLean, 1999; MacLean, 2016). This single category of budworm defoliation (moderate and severe combined) is the only spatially explicit measure of budworm incidence available on the national scale, and so more detailed explorations of the effects of low, moderate, and severe defoliation were not possible. We generated two variables representing the area affected by budworm defoliation at a local (route) level in a given year and at the stratum level in the previous 4 years. In a GIS, we overlaid the defoliation data with the road path of each BBS route. The BBS is a road‐side survey where 50 point counts (3‐min duration each) are conducted at approximately 800‐m intervals along a randomly designated route. The road paths represent the most precise spatial information currently available on BBS routes for the years in our study (available at: https://www.mbr-pwrc.usgs.gov/bbs/). The route‐level budworm variable was calculated as the proportion of the area within a 5 km buffer of each BBS route‐*j*, in stratum‐*i*, that was affected by moderate or severe defoliation in each year‐*t* ($b_{1_{i,r,t}}$). We chose a 5 km buffer as a partly arbitrary, but purposefully broad, scale that would compensate for errors in both spatial datasets, as well as variation among political regions, in the spatial resolution at which the budworm data are mapped (MacLean & MacKinnon, 1996). At a broader scale, the stratum‐level budworm variable was calculated for each year‐*t*, as the average, across the previous 4 years, of the proportion of the forested area of each stratum‐*i* that was affected by moderate or severe defoliation ($b_{2_{i,t}}=\sum_{y=1}^{4}b_{2_{i,t-x}}/4$). A 4‐year lag was chosen because local responses to budworm by warblers can continue over 4–5 years (Venier et al., 2009), and the effect of prolonged defoliation on forest health relates to the defoliation over the last 4–5 years (Erdle & MacLean, 1999; Gray & MacKinnon, 2006; MacLean, 2016).

### 2.4 Statistical modeling

The BBS count data were modeled as overdispersed Poisson variables and hierarchical Bayesian models were fit using Markov Chain Monte Carlo methods in JAGS (Lunn, Spiegelhalter, Thomas, & Best, 2009) implemented through R with package rjags (Plummer, 2016). With the exception of the budworm defoliation covariates, the model is very similar to the first difference model described in Link and Sauer (2016). Hierarchical Bayesian approaches are well suited for population time‐series analyses because they provide a robust framework to account for sources of variation at multiple scales including observer effects, overdispersion, and the scale‐ and year‐specific effects of budworm defoliation. We also included terms to account for variation in warbler species abundance among route–observer combinations, and strata, as well as a term to account for bias in counts during an observer's first year surveying a given BBS route (Link & Sauer, 2002).

In the hierarchical model, for a given species, the count of birds observed on route‐*j*, in stratum‐*i*, in year‐*t* is a random Poisson variable (note: Models were run separately for each species), where:

$$c_{i,j,t}=\text{Poisson}\left(\lambda_{i,j,t}\right)$$

The means of the Poisson distributions (λ_*i*,*j*,*t*_) are functions of the following parameters: a year effect (γ_*i*,*t*_), a combined observer–route effect (ω_*j*_), the effect of an observer's first year on a given route (ζ**I**_2_(*j*, *t*)), where **I**_2_(*j*, *t*) is equal to 1 if year‐*t* is the first year of data for observer–route combination‐*j*, and 0 otherwise), an observation‐level random effect that accounts for overdispersion (ε_*i*,*j*,*t*_); a local, route‐level budworm effect ($\beta_{1_{i}}$); and, a regional, stratum‐level, time‐lagged budworm effect ($\beta_{2_{i}}I_{1}\left(t\right)$, where **I**_1_(*t*) is equal to 1 if year‐*t* is >1969). We included the indicator variable **I**_1_(*t*) so that we could include data from the very earliest years of the BBS survey (1966 onwards), despite the fact that budworm data were only available going back to 1966, and so 1970 was the first year for which we could calculate the regional defoliation. The full equation was as follows:

$$\log\left(\lambda_{i,j,t}\right)=\gamma_{i,t}+\beta_{1_{i}}\times b_{1_{i,j,t}}+\beta_{2_{i}}I_{1}\left(t\right)\times b_{2_{i,t}}+\omega_{j}+\zeta I_{2}\left(j,t\right)+\epsilon_{i,j,t}$$

The budworm parameters for each stratum were treated as random effects, drawn from distributions with common means (*B*_1_, *B*_2_) and variances ($\sigma_{B_{1}}^{2},\sigma_{B_{2}}^{2}$), where:

$$\beta_{1}∼N\left(B_{1},\sigma_{B_{1}}^{2}\right)$$

$$\beta_{2}∼N\left(B_{2},\sigma_{B_{2}}^{2}\right)$$

These hyperparameter means (*B*_1_, *B*_2_) represented the average effects of budworm (local and regional, respectively) on species abundance across all strata.

The year effects were modeled as a first difference time series γ_*i*,*t*_, estimating the log‐scale abundance in year‐*t* and stratum‐*i* as a function of the abundance in the previous year. So for all years where *t* > 1,

$$\gamma_{i,t}=\text{Normal}\left(\gamma_{i,t-1},\sigma_{\lambda }^{2}\right)$$

And for year *t* = 1,

$$\gamma_{i,t}=\text{Normal}\left(0,\sigma_{\lambda }^{2}*1000\right)$$

The observer effect and overdispersion parameter were modeled as mean‐zero, normally distributed, random effects with estimated variances ($\omega_{j}∼N\left(0,\sigma_{\omega }^{2}\right)$ and $\epsilon_{i,j,t}∼N\left(0,\sigma_{\epsilon }^{2}\right)$). We calculated the 95% credible intervals for parameter estimates by estimating 2.5 and 97.5 quantiles from the posterior distribution of each parameter. In addition, to provide an easily interpretable measure of response to budworm, the local and regional effects are presented as estimates of percent change in warbler abundance when the routes and strata are affected by defoliation, calculated as exponents of log‐scale coefficients β and B, for example, a β value of 1.1 was transformed to exp(1.1)*100 = 300, representing a 300% increase in warbler abundance.

### 2.5 Population trajectories

To visualize modeled effects of budworm on warbler abundance, we generated three predicted population trajectories for each warbler species. Annual values in these trajectories (*N*_*i*,*t*_) represented the predicted mean count of each species in stratum‐*i* and year‐*t*, averaged across observers and routes in the stratum. The three trajectories were as follows: (i) the budworm trajectory ($N_{b_{i,t}}$)—representing the predicted abundances from the model including the modeled effect of budworm; (ii) the no‐budworm trajectory ($N_{0_{i,t}}$)—representing the predicted abundances from the model after removing the modeled effect of budworm (i.e., what would the species trajectory have been without the budworm outbreak); and (iii) the naïve trajectory ($N_{n_{i,t}}$)—representing the predicted abundances from a separately fit, naïve model that only considered the warbler data and contained no‐budworm information (with parameters $\gamma_{i,t}^{\prime },\omega_{i}^{\prime }$, and $\sigma_{\epsilon }^{\prime 2}$ estimated from a separate first difference time‐series model, identical to the original model but without the β parameters or the budworm predictors). The three trajectories were calculated as follows, where o_i_ represents the subset of observer–route combinations (O) that occur in stratum‐*i*,* r*_*i*_ is the number of observer–route combinations in stratum‐*i*, and *z*_*i*_ is the proportion of routes in stratum‐*i* on which the species has been observed (following Link & Sauer, 2002):

$$N_{b_{i,t}}=z_{i}\times \left(\frac{\sum_{j\in o_{i}}^{o_{i}\subset O}e^{\gamma_{i,t}+\beta_{1_{i}}\times b_{1_{i,j,t}}+\beta_{2_{i}}I_{1}\left(t\right)\times b_{2_{i,t}}+\omega_{j}+0.5\times \sigma_{\epsilon }^{2}}}{r_{i}}\right)$$

$$N_{0_{i,t}}=z_{i}\times \left(\frac{\sum_{j\in o_{i}}^{o_{i}\subset O}e^{\gamma_{i,t}+\omega_{j}+0.5\times \sigma_{\epsilon }^{2}}}{r_{i}}\right)$$

$$N_{n_{i,t}}=z_{i}\times \left(\frac{\sum_{j\in o_{i}}^{o_{i}\subset O}e^{\gamma_{i,t}^{\prime }+\omega_{j}^{\prime }+0.5\times \sigma_{\epsilon }^{\prime 2}}}{r_{i}}\right)$$

Comparing the budworm versus no‐budworm trajectories allowed assessment of the effect of budworm outbreaks on predicted values, whereas comparing budworm against naïve trajectories allowed assessment of how much of the underlying temporal variation was captured by the model incorporating budworm effects.

## 3 RESULTS

**Figure 2:**
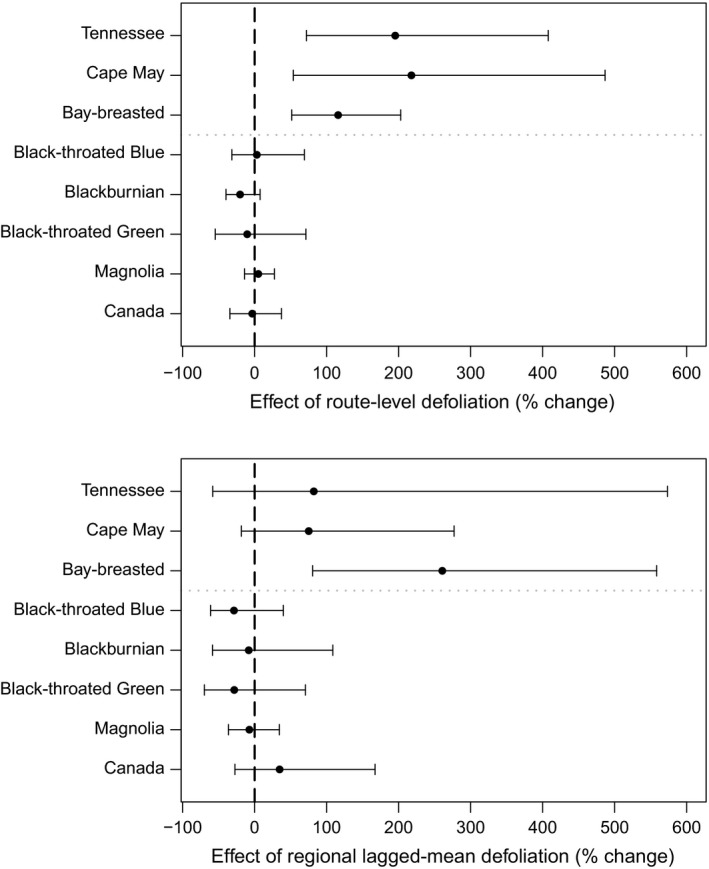
Local and regional responses of eight forest warbler species to spruce budworm in eastern Canada. Effects are estimates of percent change in mean Breeding Bird Survey (BBS) counts to attributed spruce budworm outbreaks, based on parameters *B*_1_ and *B*_2_. Spruce budworm was measured at the local scale (upper panel) by the proportion of the area affected by severe to moderate spruce budworm defoliation within a 5 km buffer around a BBS route, and at the regional scale (lower panel) by the 4‐year average proportion of the area affected by severe to moderate spruce budworm defoliation, within a BBS geographic stratum. Bars around parameter estimates represent 95% credibility intervals. The three species above the horizontal dotted line have a known positive association to spruce budworm abundance

**Figure 3:**
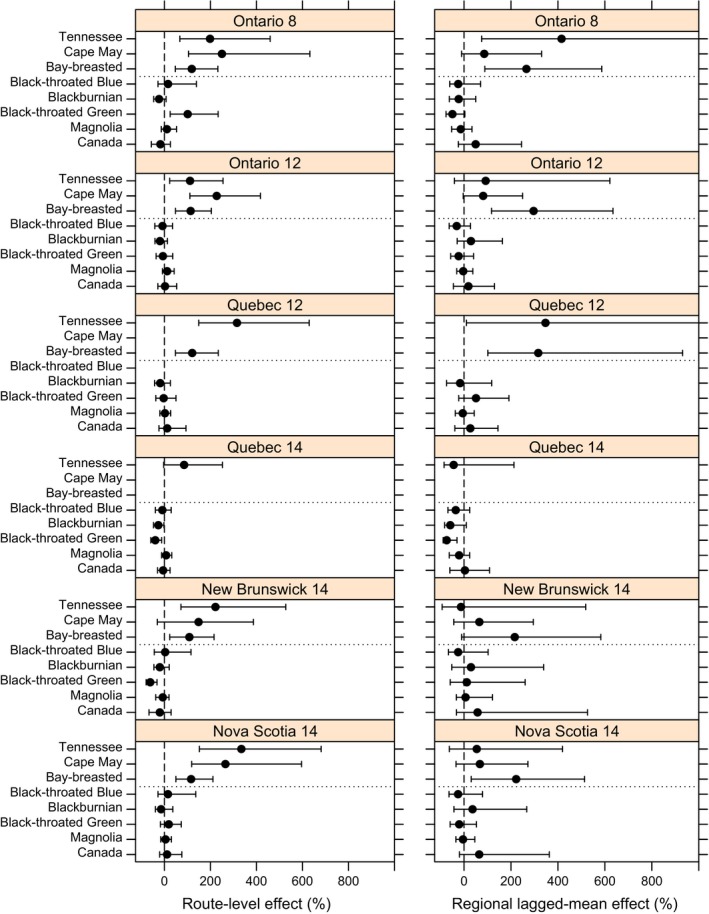
Stratum‐specific responses of eight forest warbler species to spruce budworm in eastern Canada. Effects are estimates of percent change in mean Breeding Bird Survey (BBS) counts to spruce budworm outbreaks within a geographic stratum. Spruce budworm was measured at the local scale (left column of panels) by the proportion of the area affected by severe to moderate spruce budworm defoliation within a 5 km buffer around a BBS route, and at the regional scale (right column of panels) by the 4‐year average proportion of the area affected by severe to moderate spruce budworm defoliation, within a BBS geographic stratum. Bars around parameter estimates represent 95% credible intervals. The three species above the horizontal dotted line have a known positive association to local spruce budworm abundance

At the route level, all three budworm‐associated warblers showed strong positive responses to the local budworm defoliation both averaged across all strata (*B*_1_, Figure 2) and within strata ($\beta_{1_{i}}$, Figure 3). Estimates of percent change indicated that counts of Tennessee and Cape May Warblers increased by ~200% when 100% of the 5 km buffer around a BBS route was affected by defoliation, and bay‐breasted warblers increased by >115% (Figure 2). Within each stratum, results for the three budworm‐associated warblers were similar to the overall response, showing strong positive effects with estimates of percent change ranging from 85% to 334% (Figure 2). The other five warbler species showed muted responses to local budworm defoliation, with hyperparameter percent change estimates for route‐level effects ranging from −20% to 5% (Figure 2), with 95% credible intervals that overlapped with zero. Stratum‐specific percent change estimates were similar in direction and magnitude across all strata for the non‐budworm‐associated species (Figure 3), except for black‐throated green warbler where responses were positive in some strata and negative in others.

At the regional level, bay‐breasted warbler showed a strong positive response to regional budworm defoliation averaged across the previous 4 years, both overall (i.e., averaged across all strata; *B*_2_, Figure 2) and within each of the strata ($\beta_{2_{i}}$, Figure 3). The percent change coefficients suggest that counts of bay‐breasted warblers on all BBS routes in a region increased by 260% when the region had been affected by defoliation over the previous 4 years, in addition to any increases due to the local budworm defoliation on individual routes. The point estimates (i.e., means of the posterior distributions) of overall regional responses for the other two budworm‐associated species were also positive and were approximately 80% (Figure 2), although credible intervals overlapped with zero. For Tennessee warbler, stratum‐level effects were a mix; strong and positive in two strata, weakly positive in two, and weakly negative in two (Figure 3). For Cape May warbler, stratum‐level effects were all positive and ranged between 65% and 86%, but were sufficiently imprecise that their credible intervals included zero in all cases (Figure 3).

The remaining five species showed no clear regional‐level response to budworm defoliation. Point estimates for regional‐level percent change were negative and ranged from −7% to 29% (Figure 3), but with credible intervals that included zero, and stratum‐level responses were a mix of weakly positive and negative responses (Figure 3). The exception was Canada warbler, which had a positive estimate for overall mean regional response of 30%, particularly in New Brunswick and Nova Scotia, and all positive stratum‐level estimates ranging from 3% to 57%, however, all with credible intervals that included zero (Figure 3).

### 3.1 Population trajectories

**Figure 4:**
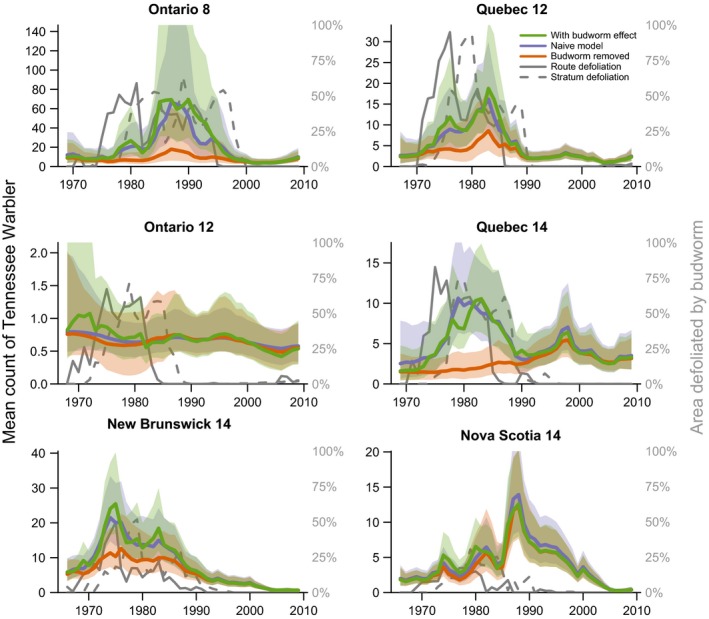
Three predicted population trajectories for Tennessee warbler in six geographic strata (values indicated by the left‐side axes) and trajectories for the two spruce budworm defoliation predictors used in the models (values indicated by the right‐side axes). The green trajectories show the predicted average annual counts of birds on BBS routes in the region, including the effects of the budworm. The orange trajectories show the predicted counts after removing the estimated effect of the budworm. The blue trajectories provide a model‐checking comparison to the green trajectories, showing the predicted counts from a naïve model with no‐budworm information. Note: The route‐level defoliation values are plotted as the average across all routes for a given year, although the model used values specific to each BBS route. Polygons around trajectories indicate 95% credible intervals

**Figure 5:**
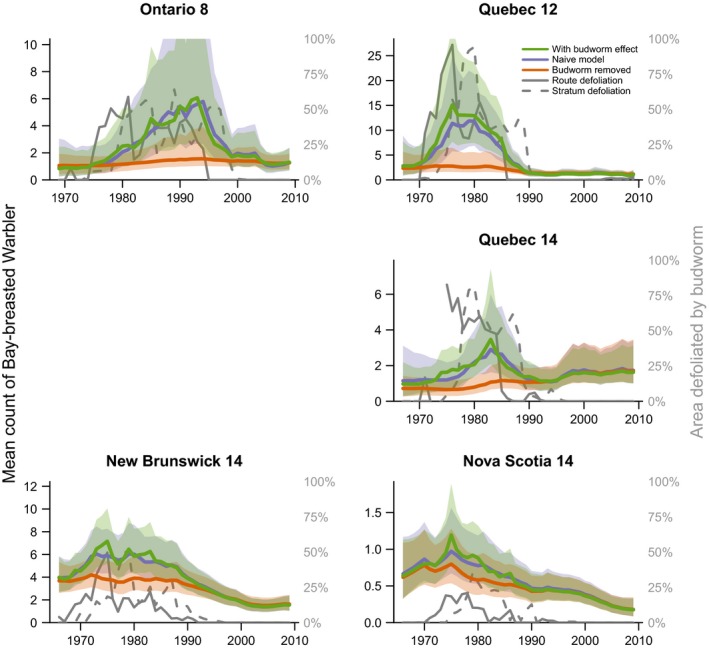
Three predicted population trajectories for bay‐breasted warbler in six geographic strata (values indicated by the left‐side axes) and trajectories for the two spruce budworm defoliation predictors used in the models (values indicated by the right‐side axes). Trajectories as in Figure 4 caption

**Figure 6:**
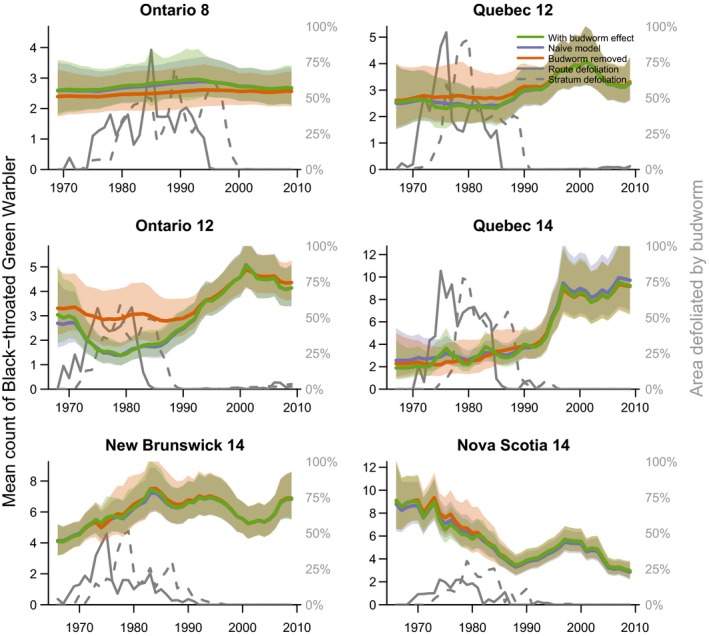
Three predicted population trajectories for black‐throated green warbler in six geographic strata (values indicated by the left‐side axes) and trajectories for the two spruce budworm defoliation predictors used in the models (values indicated by the right‐side axes). Trajectories as in Figure 4 caption

**Figure 7:**
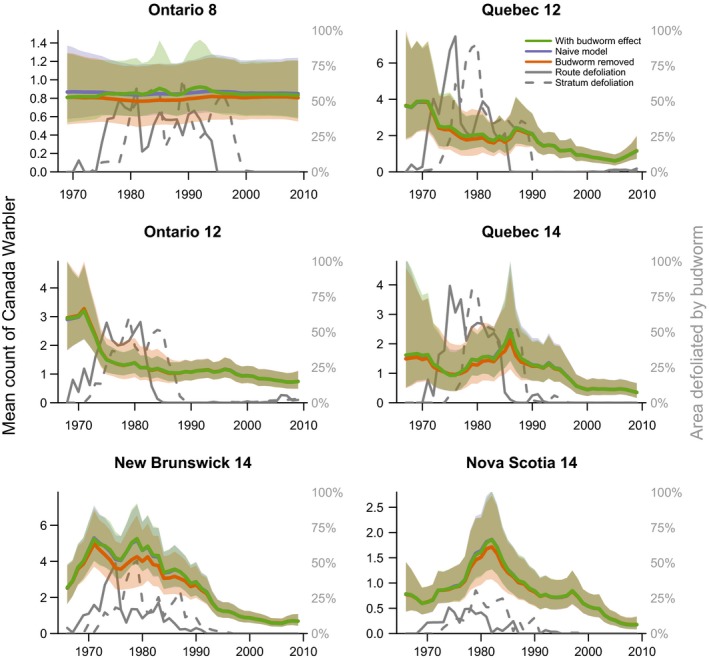
Three predicted population trajectories for Canada warbler in six geographic strata (values indicated by the left‐side axes) and trajectories for the two spruce budworm defoliation predictors used in the models (values indicated by the right‐side axes). Trajectories as in Figure 4 caption

The predicted population trajectories for the budworm‐associated species demonstrate the magnitude of species responses to budworm outbreaks in the 1970s and 1980s (Figure 4 Tennessee warbler, and Figure 5 for bay‐breasted warbler; Cape May warbler trajectories are included in the Supporting information Appendix S1). The strongest effects (i.e., the largest differences between the “with budworm” (green color) and “no‐budworm” (orange color) trajectories) were in the three strata where overall levels of defoliation were highest and where the warblers were most abundant overall (i.e., Ontario‐8, Quebec‐12, and Quebec‐14). In these strata, the budworm outbreaks of the 1970s and 1980s were likely responsible for sixfold to 12‐fold increases in regional populations of these three warbler species. In the other strata, the outbreaks resulted in smaller increases in regional populations, in part because of the generally smaller scale of the outbreaks (compare the defoliation (gray solid and dashed lines) among strata). Comparisons between trajectories with budworm (in green) and naïve‐model trajectories (in blue) showed that predictions from the budworm model were similar to predictions from the population status and trend model typically used to analyze the BBS counts (e.g., a first difference model similar to that used by Link & Sauer, 2016), indicating that the spruce budworm model well characterized the variation in abundances of these three species. Trajectories for the five other species varied, but in each case concordance between predictions from the naïve and budworm models indicated the budworm model had well characterized the temporal variation in warbler counts (Figures 6 and 7 for trajectories of black‐throated green and Canada warbler; all other species in the Supporting information Appendix S1).

The population trajectories also allowed us to identify geographic variation that may exist in how warbler responded to budworm outbreaks, even for species that showed an overall dampened response. For example, comparing predictions from the budworm effect to the no‐budworm model indicated that a depression in counts of black‐throated green warblers in the Ontario‐12 stratum during the 1970s and 1980s coincided with the budworm outbreak (Figure 6). Similarly, fluctuations in abundance of Canada warblers in Ontario strata occurred independently of the budworm outbreak, in contrast to the high counts of Canada warbler that occurred in the New Brunswick‐14 stratum between 1970 and 1990, and coincided directly with the outbreak (Figure 7).

## 4 DISCUSSION

Warblers showed both local and regional responses to spruce budworm outbreaks in eastern Canada. Our modeling approach incorporated effects of budworm directly on the warbler count data and improves on previous iterations that calculated correlations between budworm defoliation with derived indices of bird abundance (Sleep et al., 2009; Venier et al., 2012). More importantly, it allowed us to simultaneously evaluate correlations between warbler abundance and budworm defoliation at two different spatial scales in the same model. We compared the population‐level responses to spruce budworm among species that differed in their known associations with spruce budworm and found that the model performed well under a range of situations. Responses varied by species, with budworm‐associated species responding strongly to both local and regional effects, and response of other species being limited to regional effects which varied across strata. These differences underscore the complexity of forest bird responses to insect outbreaks, which may involve both increased numerical responses to food supply, and longer term responses to changes to forest structure and composition, for example, black‐throated green warbler showed positive local response and negative regional response in BCR 8 Ontario. Because the model simultaneously estimates both local and regional effects, this approach can resolve some of the uncertainties associated with such scale effects (Venier & Holmes, 2010), and may be readily applied to other disturbance events, such as fire, that have immediate and lagged regional effects on forested landscapes.

Budworm‐associated species showed weaker regional responses in the Atlantic Provinces than in Ontario or Quebec, and such strata‐scale differences in responses may be related to variation in both forest management and the overall scale of the outbreak among provinces. In both New Brunswick and Nova Scotia, the defoliation had relatively lower spatial extents and very rarely exceeded 25% of the region (3 years in New Brunswick, and 2 years in Nova Scotia), while in the other regions the peak in regional defoliated area ranged from 55%–95% of the region and exceeded 25% in 12–17 years (Figures 3, 4, 5, 6, 7). In New Brunswick, insecticide spraying moderated spruce budworm defoliation and tree mortality; an average of 2 million hectares per year was sprayed from 1970 to 1983 to prevent extensive tree mortality (Kettela, 1975; Webb & Irving, 1983). Forest protection policy in New Brunswick specified that stands had to sustain moderate to severe defoliation for at least two successive years before they became eligible for insecticide spraying, resulting in periodic defoliation, and insecticide spraying under this policy was generally effective in keeping trees alive (Virgin & MacLean, 2017). In Nova Scotia, the outbreak was largely confined to Cape Breton Island and the north‐central part of the province, where heavy tree mortality occurred (MacLean & Ostaff, 1989). Nova Scotia forests have higher proportions of red spruce, which is less vulnerable than the balsam fir which dominates in New Brunswick (MacLean, 1980; MacLean, Beaton, Porter, MacKinnon, & Budd, 2002). As such, this province experienced reduced defoliation, with peak values of ~25% in contrast to the other strata where defoliation peaked at 75%–100% (Figures 4, 5, 6, 7). These differences in forest overstory composition and moderation of budworm‐caused mortality by insecticide spraying in the smaller Atlantic Provinces contrasted with Quebec and Ontario, where stronger regional responses by warblers occurred and less pervasive insecticide spraying was conducted. These factors would result in different composition of the residual canopy of nonhost trees, and might affect responses by birds, or create differences among strata between defoliation and “unmeasured” food supply, that is, the correlations between defoliation and larval abundance may vary somewhat among provinces, especially during the early stages of an outbreak.

The positive association between some warblers and spruce budworm indicates that a trade‐off may exist between timber supply and benefits to forest birds, wherein management agencies must decide whether to keep outbreaks under control or allow outbreaks some opportunity to act as a natural disturbance agent on the landscape. From a silvicultural perspective, spruce budworm outbreaks may emulate shelterwood regeneration cuttings in mature stands and thinning operations in pole‐size stands (Baskerville, 1975), and thus contribute to the uneven‐aged stand management regimes associated with higher biodiversity values (Drever et al., 2006). However, if bird community shifts (increased populations of 1 or 2 species) result in competitive disadvantage or exclusion for other species, then budworm outbreaks could be viewed as conservation‐neutral, for example, we found that budworm outbreaks were associated with reduced numbers of Blackburnian and black‐throated green warblers in Quebec and New Brunswick, which may be attributable to the incursion of aggressive species like the bay‐breasted warbler (Morris et al., 1958). Such competitive interactions may form part of the natural repercussions of pulses in food supply and will need to be well understood as forest management regimes strive to emulate natural disturbances. Determining the full effect of spruce budworm outbreaks will require an assessment of the entire bird community (Venier et al., 2009), and the modeling approach presented here could be expanded to evaluate local and regional effects on a wide range of species, as well as interactions among species.

Abundance of spruce budworm will likely increase in eastern Canada in the coming years. Spruce budworm outbreaks are cyclical, and every 30–40 year populations increase to outbreak levels where they remain for 10 years or longer. From the perspective of the warblers, spruce budworm outbreaks act as a resource pulse (Yang, Bastow, Spence, & Wright, 2008) that provides a superabundant food supply that moves across the landscape in complex ways. We are the first to study the response of warblers to budworm at multiple scales simultaneously. This cross‐spatial analysis provides population‐level inferences across a large study area spanning wide variation in forest composition and management practices, and identifies a regional response after controlling for a local response, even for the three warbler species for which a local response had been well established. The population‐level responses to budworm outbreaks estimated here will be particularly useful to inform the conservation management of some warblers that have shown large decreases in their national populations since the 1970s (Environment and Climate Change Canada, 2017), including Canada warbler, which is listed as a species at risk in Canada because of steep long‐term population declines (COSEWIC, 2008). For Canada warbler specifically, our results demonstrate that this species is not positively associated with budworm outbreaks in the same manner as other warbler species (e.g., Cape May warbler). Our results have identified some intriguing possible relationships at the regional scale in New Brunswick and Nova Scotia, where the outbreaks were less severe than in other regions, and recent studies highlight the importance of larger landscape matrix to Canada Warbler dynamics (Grinde & Niemi, 2016). If Canada warblers have less direct or more complex relationships with spruce budworm, for example, neutral responses to increased food but positive responses to the changing forest structure, then these regions should be a good site for future work. Ultimately, our work clearly shows that population declines for Canada warbler will not be mitigated by a future budworm outbreak and that conservation efforts directed at the species and others like it must continue to consider factors across the species’ full life cycle.

## AUTHOR CONTRIBUTIONS

MCD, ACS, LAV, DJHS, and DAM conceived the ideas; LAV and DAM collected the data; ACS designed methodology and analyzed the data; MCD, ACS, LAV, DJHS, and DAM led the writing of the manuscript. All authors contributed critically to the drafts and gave final approval for publication.

## CONFLICT OF INTEREST

None declared.

## DATA ACCESSIBILITY

Breeding bird survey is publicly available and downloadable at https://www.pwrc.usgs.gov/BBS/RawData/. Defoliation data is available through the provincial governments of Ontario, Quebec, Nova Scotia, and New Brunswick.
