# Supplementary material for: Cross‐scale effects of spruce budworm outbreaks on boreal warblers in eastern Canada
Source: Ecol Evol. 2018 Jun 27;8(15):7334–45. doi: 10.1002/ece3.4244 (PMC6106201; doi:10.1002/ece3.4244)

SUPPLEMENTARY MATERIAL

Three predicted population trajectories for four warbler species (Cape May, black−throated blue, Blackburnian, Magnolia) in six geographic strata (values indicated by the left-side axes), and trajectories for the two spruce budworm defoliation predictors used in the models (values indicated by the right-side axes). The green trajectories show the predicted average annual counts of birds on BBS routes in the region, including the effects of the budworm. The orange trajectories show the predicted counts after removing the estimated effect of the budworm. The blue trajectories provide a model-checking comparison to the green trajectories, showing the predicted counts from a naïve model with no budworm information. Note: The route-level defoliation values are plotted as the average across all routes for a given year, although the model used values specific to each BBS route. Polygons around trajectories indicate 95% credible intervals.

Cape May warbler


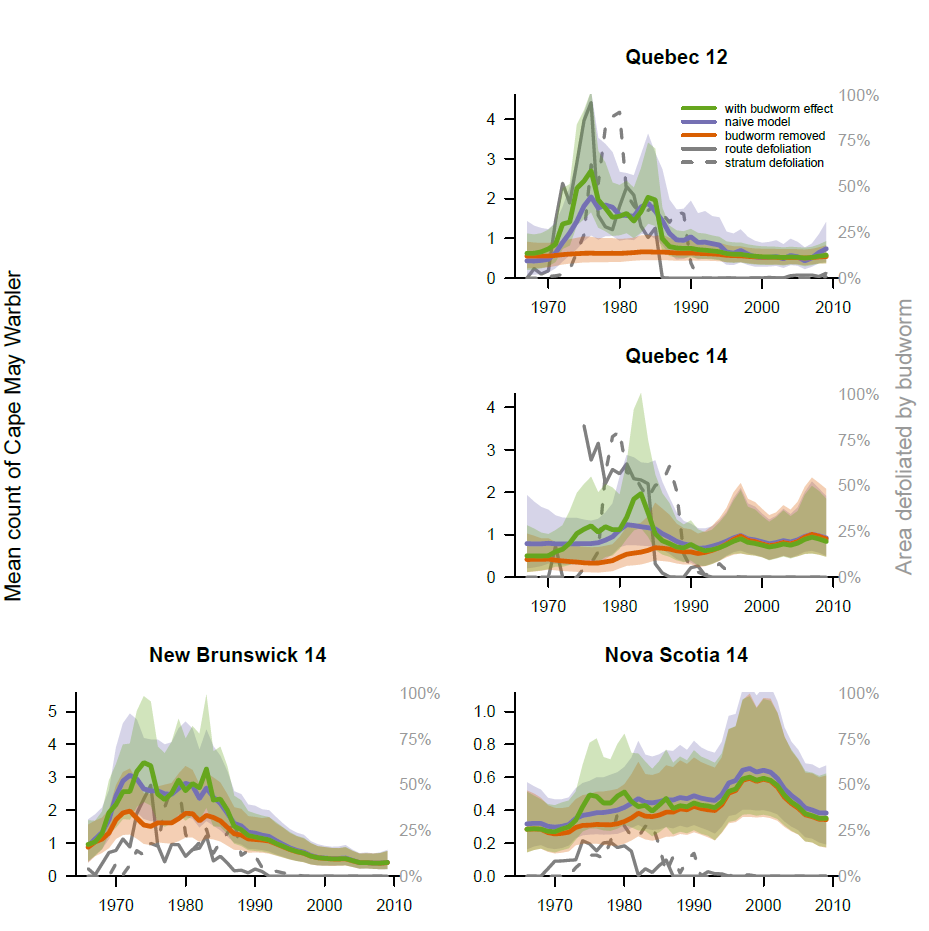


black−throated blue warbler


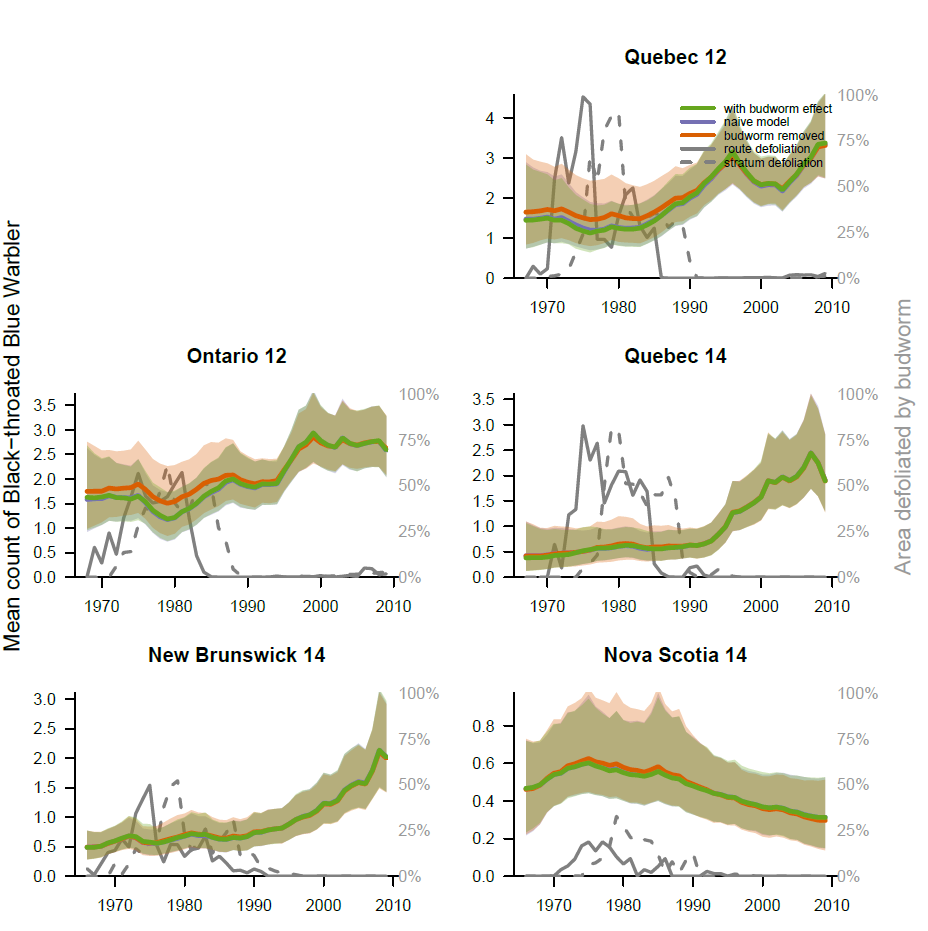


Blackburnian warbler


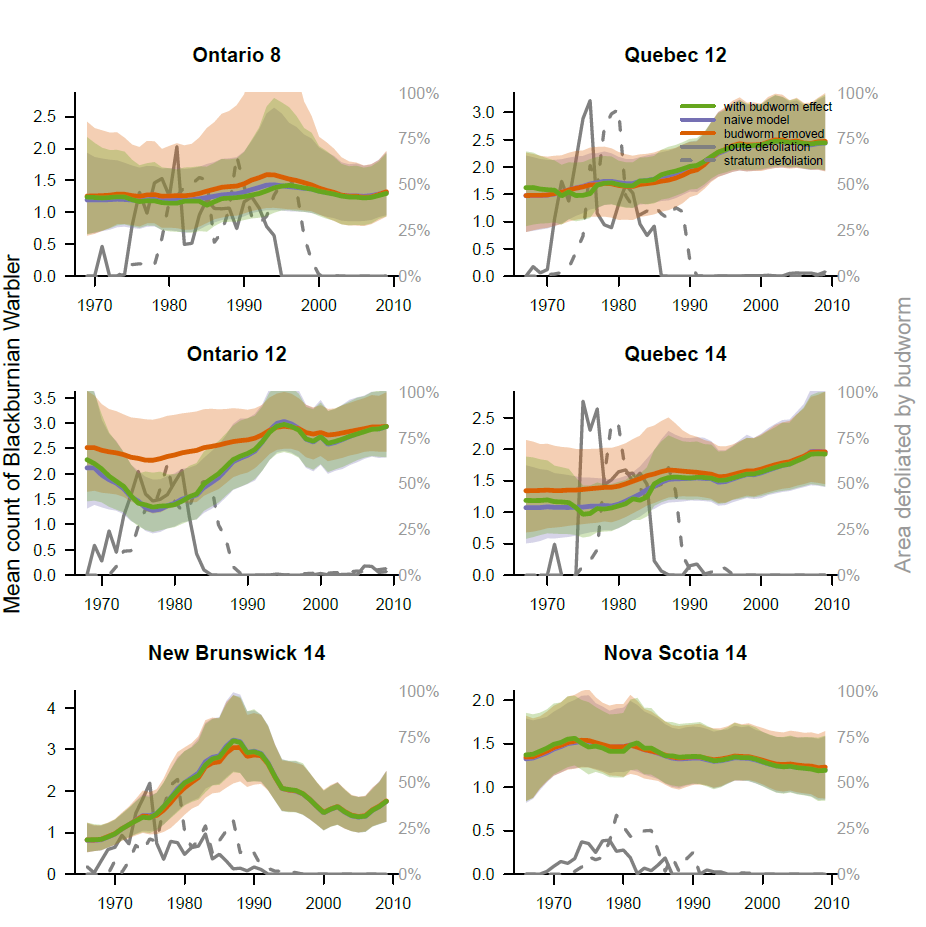


Magnolia warbler


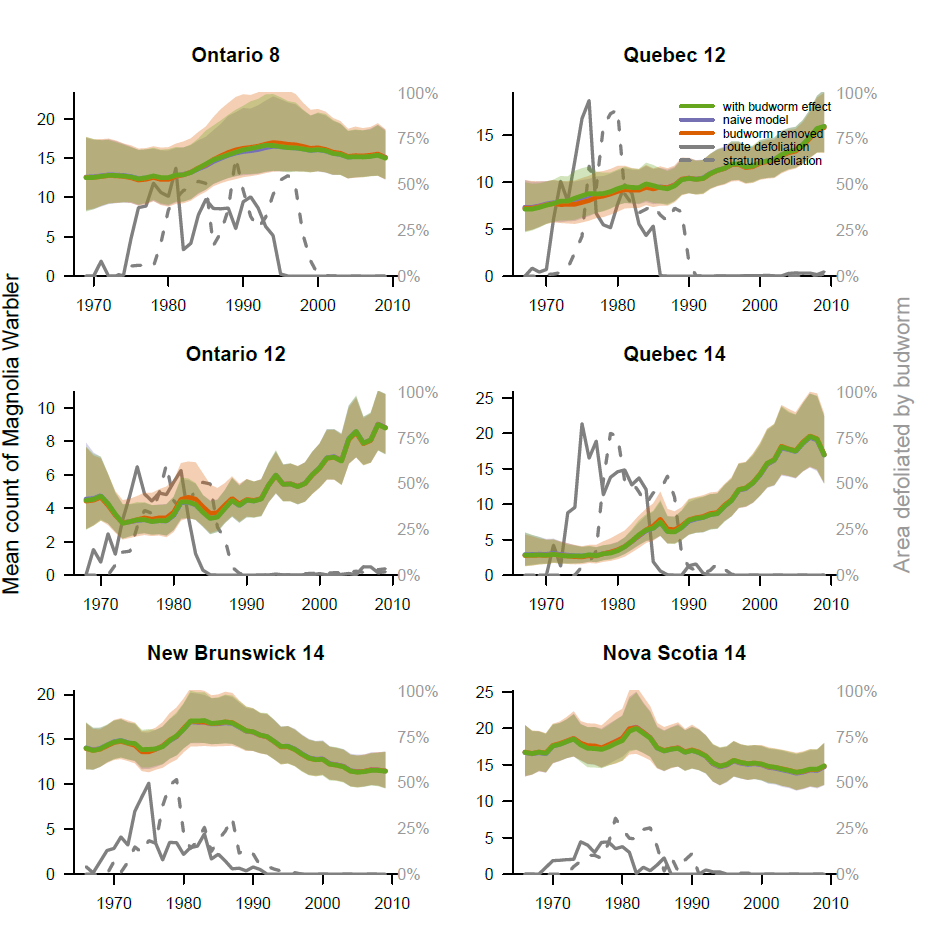

Supplement: Supplementary file 1 [file ECE3-8-7334-s001.docx]
